# Audit of Patients Prescribed Psychotropic Medication in the Community Learning Disabilities Psychiatry Services of the Black Country Healthcare NHS Foundation Trust

**DOI:** 10.1192/bjo.2024.336

**Published:** 2024-08-01

**Authors:** Adeniyi Adetoki

**Affiliations:** Black Country Healthcare NHS Foundation Trust, Dudley, United Kingdom

## Abstract

**Aims:**

Stopping Overmedication of People with a Learning Disability, Autism or Both (STOMP) is an initiative of NHS England. This was in response to concerns raised as a result of the Winterbourne View scandal related to the inappropriate use and insufficient arrangements for the review of the prescription of psychotropic medication.

33,000–35,000 individuals with an intellectual disability (ID) are prescribed psychotropic medication daily. 20–45% are on antipsychotic medication, of which 14–30% take these to control behaviour problems rather than for specified psychiatric conditions. Psychotropic medications can have side effects with the potential to significantly impair an individual's quality of life.

This audit is to observe current practice of the prescription of psychotropic medication, with a view to identifying changes to the compliance with recommendations and outlining areas for further improvement in line with the Stopping Overmedication of People with a Learning Disability, Autism or Both (STOMP) initiative.

**Methods:**

Data was collected from electronic records for randomly selected patients, 20 from each of the 4 Community Learning Disabilities Locality Teams within the Trust. The patients who were not currently prescribed psychotropic medication were excluded from the randomly selected samples.

**Results:**

There was good evidence that capacity, consent and best interests were considered, as well as multidisciplinary input. There was also good evidence of regular review of medication, side effects and treatment response. The results suggests that psychotropic medication continues to play a significant role in the management of patients presenting with behavioural problems, and more needs to be done to identify approaches that will help to reduce their use.

**Conclusion:**

In this patient group it is sometimes the case that medication is prescribed legitimately for indications other than their British National Formulary (BNF) recommended use. However, the findings suggest that the rationale could be more clearly recorded. Close collaboration with primary care to provide a comprehensive medication history, the involvement of carers and family members in the active preparation for effective medication reviews and the involvement of the multidisciplinary team should continue to be encouraged and clearly recorded.

